# Keep Your Head Clean

**Published:** 1892-11

**Authors:** 


### Keep Your Head Clean.

Keeping the head perfectly clean is a great aid to health. A distinguished physician, who had spent much of his time at quarantine, said that a person whose head was thoroughly washed every day rarely took contagious diseases, but when the hair was allowed to become dirty and matted, it was hardly possible to escape infection. Many persons find speedy relief for nervous headache by washing the head thoroughly in weak soda water. We have known cases almost wholly cured in ten minutes by this simple remedy. A friend finds it the greatest relief in cases of "rose cold," the cold symptoms entirely leaving the eyes after the first washing of the hair. The head should be thoroughly dried afterward, and draughts of air should be avoided for a little while.

